# Working with public contributors in Parkinson's research: What were the changes, benefits and learnings? A critical reflection from the researcher and public contributor perspective

**DOI:** 10.1111/hex.13914

**Published:** 2023-11-21

**Authors:** Fiona E. Lithander, Emma Tenison, David Ashford Jones, Sue Stocker, Noreen Hopewell‐Kelly, Andy Gibson, Carmel McGrath

**Affiliations:** ^1^ Ageing and Movement Research Group, Population Health Sciences Bristol Medical School, University of Bristol Bristol UK; ^2^ Liggins Institute University of Auckland Auckland New Zealand; ^3^ Department of Nutrition and Dietetics University of Auckland Auckland New Zealand; ^4^ Older People's Unit Royal United Hospitals Bath NHS Foundation Trust Bath UK; ^5^ Cure Parkinson's London UK; ^6^ Parkinson's UK London UK; ^7^ Divison of Population Medicine Cardiff University Cardiff UK; ^8^ People in the Health West of England University of the West of England Bristol UK; ^9^ The National Institute for Health and Care Research Applied Research Collaboration West (NIHR ARC West) University Hospitals Bristol and Weston NHS Foundation Trust Bristol UK; ^10^ Faculty of Health and Applied Sciences, School of Health and Social Wellbeing University of West England Bristol UK; ^11^ NIHR Health Protection Research Unit in Behavioural Science and Evaluation, Population Health Sciences Bristol Medical School, University of Bristol Bristol UK

**Keywords:** critical reflections, feedback, impact of public involvement, Parkinson's disease, patient and public involvement, Public Involvement Impact Logs, recommendations

## Abstract

**Introduction:**

This paper provides a critical reflection from both the researcher and public contributor (PC) perspective on the benefits and the learnings taken from involving PCs in research related to Parkinson's.

**Approach to Patient and Public Involvement (PPI):**

This paper reports on how PCs shaped the design and development of the PRIME‐UK research programme study materials through input into information leaflets, consent forms and other patient‐facing documents used across three studies within the PRIME‐UK research programme. The PRIME‐UK research programme is designed to improve the quality of life of people with Parkinson's and this project included three studies: a cross‐sectional study, a randomised control trial and a qualitative study. We captured these impacts using Public Involvement Impact Logs, which provide a framework allowing researchers and PCs to report on the learnings, immediate outcomes and impacts from PPI. For this project, the impact logs enabled us to provide reflections from PCs and researchers on the process of involving ‘the public’ in Parkinson's research.

**Findings:**

This paper builds on existing evidence of the range of benefits and challenges that emerge from working with patients and the public in Parkinson's research; this includes reflecting on the changes made to the study materials and benefits for the people involved. Four themes emerged from the reflections that were common to the researchers and PCs; these were the importance of providing a supportive environment; recognition of the benefit of the evaluation of the impact of PPI; acknowledgement that engagement of PPI can make a positive difference to the research process and that timely communication and the use of face‐to‐face communication, where available, is key. Furthermore, we demonstrate how impact logs provide a useful and straightforward tool for evaluating public involvement practices and supporting the feedback process.

**Conclusion:**

We offer key recommendations for involving patients and the public in Parkinson's research and suggest approaches that could be implemented to capture the impacts of public involvement.

**Public Contribution:**

Public contributors (PCs) were involved in the design and development of the participant information leaflets, consent forms and other patient‐facing documents used for studies within the PRIME‐UK research programme. In addition, PCs evaluated their involvement using impact logs and co‐authored this paper.

## 1 INTRODUCTION

Patient and public involvement (PPI) is increasingly prominent within health research in the United Kingdom and internationally.^1^, ^2^ There is growing interest from research funders in developing methods to evaluate the impacts that result from working in partnership with patients and the public in research.^1^, ^3^, ^4^ Evidence has shown that PPI can improve the relevance, quality and acceptability of health research and leads to benefits for those involved and for the wider communities.^5^, ^6^, ^7^ However, there are different views on whether and how the impacts of PPI should be evaluated.^8^, ^9^, ^10^ Some believe that PPI should be embedded in research because patients and the public have a right to be involved in research, regardless of the impact.^11^, ^12^ Others consider it important to provide evidence of the tangible and practical impacts that PPI has made on research.^10^ Staley points out that the benefits of doing PPI in research are well established even though difficult to quantify, and questions whether further attempts at measuring impact are necessary or desirable.^8^ She suggests that whether public involvement is worth doing or not depends on a range of contextual factors, so the answer to this type of question will always be ‘maybe’. Furthermore, many public contributors (PCs) feel uncomfortable with the notion that their contribution to research is being singled out for evaluation.

The present authors do not see a necessary conflict between a rights‐based approach to PPI and one that emphasises the potential benefits of public involvement for the quality and relevance of research. We would agree with Staley that, in general, the benefits of PPI for research are well established but believe we need to move beyond her ‘maybe’ response. PPI is an evolving discipline and there is much to be learned about the best way to practice it in relation to diverse groups of people and with a wide range of research methodologies. We suggest that evaluations are carried out looking at the input of PCs within specific research methods and topic areas with an emphasis on improving practice so that the full potential benefits of PPI are maximised for both PCs and researchers.

While the National Institute for Health and Care Research (NIHR) encourages researchers to involve PCs collaboratively and at every stage of the research process, the researchers writing this paper were relatively new to the practice of PPI. This paper includes honest reflections and learnings from the researcher and PC perspective on conducting involvement in Parkinson's research, acknowledging where future practices could be improved to achieve more meaningful and embedded PPI.

There have been several developments in PPI impact and evaluation in recent years, for example, the inclusion of a standard on Impact in the UK Standards for Public Involvement^13^ and the development of a NIHR definition of PPI impact.^14^, ^15^, ^16^, ^17^ This defines impact as, ‘The changes, benefits and learning gained from the insights and experiences of patients, carers and the public when working in partnership with researchers and others involved in NIHR initiatives’.^14^ The definition acknowledges that, while tangible changes to research are recognised as impact, equally important are the benefits and learnings for researchers and PCs.

There are several tools available to evaluate PPI in research. Some are designed specifically with PPI in mind, for example, Public Involvement Impact Logs, the Cube Evaluation Framework and the more comprehensive Public Involvement Impact Assessment Framework.^18^, ^19^ Others have adopted methodologies drawn from the wider evaluation literature, for example, realist evaluations.^20^, ^21^

One of the difficulties with evaluating PPI is the additional work that it generates for both researchers and PCs. A Public Involvement Impact Log,^19^ developed by colleagues and PCs from the People in the Health West of England, UK (a regional PPI network based in the South West of England), was used in the current study. The Public Involvement Impact log provides a simple framework that enables both PCs and researchers to report and reflect on learnings, immediate outcomes and longer‐term impacts following any given PPI activity.^19^ The log is both a record of PPI activity and a reflective tool that can be used longitudinally throughout the research cycle as a space for both PCs and researchers to consider their own role and learning in PPI.

The existing literature has revealed that there is a small body of evidence reporting PPI practices and impacts on Parkinson's research.^15^, ^22^, ^23^ In comparison PPI has made considerable progress in areas including dementia, rheumatology and cancer research.^2^, ^24^, ^25^, ^26^, ^27^, ^28^ We hope that the insights reported in this paper will further enhance PPI practice in Parkinson's research.

In this paper, we use the NIHR definition of PPI impact to reflect on the changes, benefits and learnings gained from working with PCs on a research programme on Parkinson's.^13^, ^14^ We conclude by offering key recommendations for how to involve patients and the public in Parkinson's research and suggest approaches that could be used in practice to capture the impacts of PPI. The concluding section of this paper reflects on the use of impact logs as a framework to capture PPI impact.

## 2 APPROACHES TO PPI

### 2.1 Recruitment

PCs were recruited through Parkinson's UK and by word of mouth. While face‐to‐face sessions were planned, all communication took place by email, phone and video conference due to the advent of pandemic restrictions. There was no prior relationship between PCs and researchers before the PPI activities began as part of this programme of research. The researchers were colleagues working on the PRIME‐UK research programme and had known each other for approximately 6 months at the point the PPI activities first began. Ethical approval was not sought as it is not required for PPI activities.^29^

### 2.2 Design: Background of the PRIME‐UK research programme in Parkinson's and the PPI activities related to this

The researchers and PCs who took part in this paper were all involved in the PRIME‐UK research programme, which is designed to improve the quality of life of people with Parkinson's. PCs contributed their views on aspects of three individual studies within the PRIME‐UK research programme: (1) the PRIME‐UK cross‐sectional study, which aimed to describe the needs and experience of people with Parkinson's along with their informal caregivers^30^; (2) the PRIME‐UK randomised control trial (RCT), which is evaluating a multicomponent intervention^31^ and (3) the PRIME‐UK qualitative study, which is embedded within the RCT.

### 2.3 PC involvement in the PRIME‐UK cross‐sectional study

PCs were asked to comment on the term used to describe someone who provided informal care for a person with Parkinson's (PwP). They were also asked to review and comment on the participant information leaflets (PILs), consent form and a document entitled ‘About me’, which asks for some basic information about who, if anyone, the PwP lives with, whether they have an informal caregiver and any requirements they may have, so that these could be accommodated in the research process.

### 2.4 PC involvement in the PRIME‐UK RCT and the PRIME‐UK qualitative study

PCs were asked to comment on the PIL, consent form and treatment plan template for PwP for the PRIME‐UK RCT and the topic guides for PwP and caregivers for the PRIME‐UK qualitative study, respectively. Regarding the PILs and consent forms, they were asked to comment on the clarity of the research purpose, explanation of the study procedures and language used, and any steps the researchers could take to make it easier for PwP to take part. They were asked to provide feedback on the questions outlined in the topic guides and discuss if there was anything potentially inappropriate or missing. The researchers responded to PCs to thank them for their time and contribution and with a description of how their comments were integrated into the documents, as appropriate. If comments received were unable to be integrated, this was explained by the researchers to the PCs.

After the PCs' involvement in these activities, one of the researchers (F. E. L.) emailed each PC individually with an introduction to the evaluation of PPI and with questions related to their experience of providing their views on the three studies. PCs were subsequently contacted by one of the researchers (E. T.) to provide their reflections for this paper. Researchers (F. E. L. and E. T.) were also asked to provide their reflections on PPI in the research process.

### 2.5 Evaluation of the PPI using the Public Involvement Impact Log

**Figure 1:**
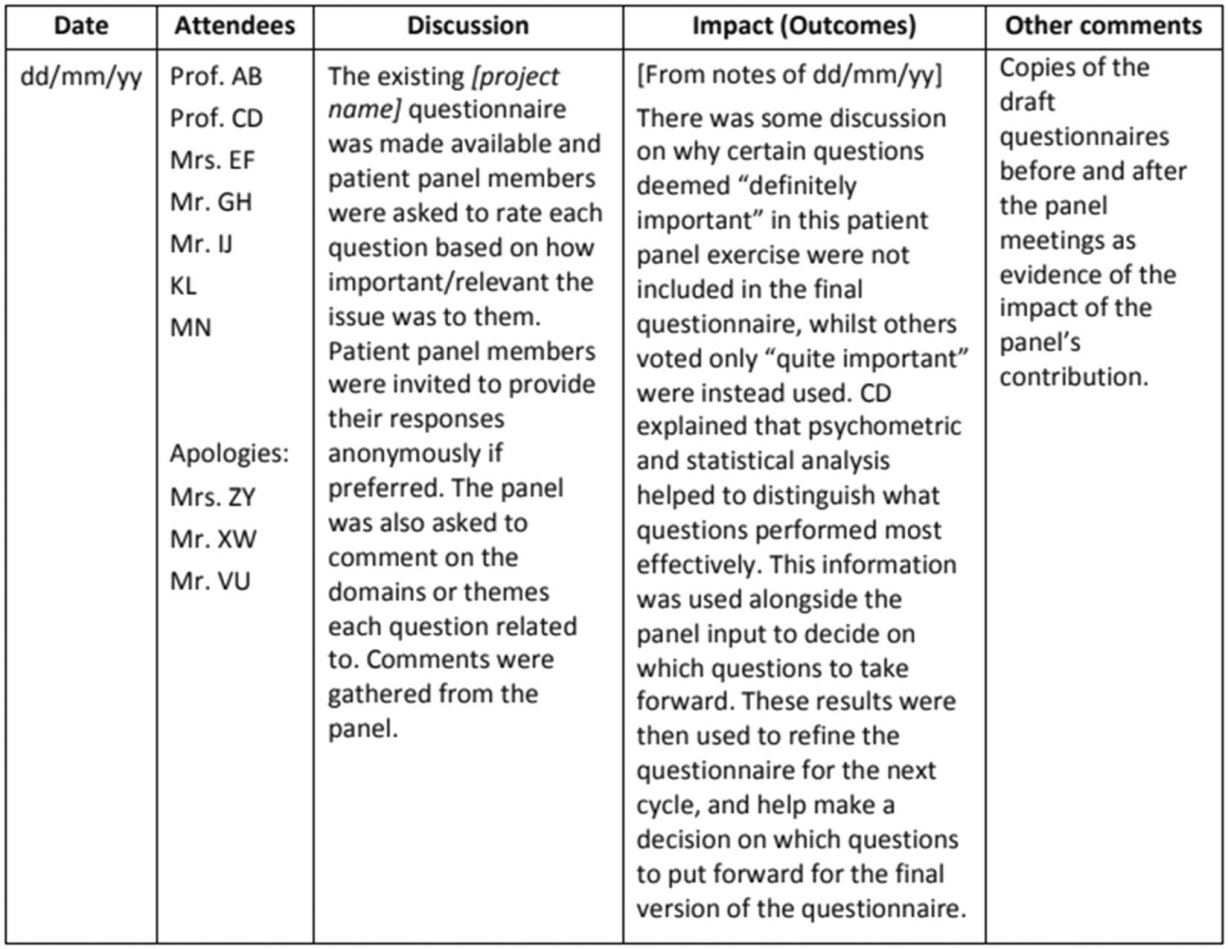
Public involvement impact Log, taken directly from Kok.^19^

The questions in the original Public Involvement Impact Log (Figure 1) were based on the findings of a systematic review that was undertaken by Health Protection Research Unit colleagues (including co‐author N. H.‐K.). The review examined frameworks of impact and evaluation in PPI. Its findings highlighted the areas that were consistently reported across papers. These formed the basis of the original questions (Figure 1), which were further refined and developed with input from a separate group of PCs. The log was intended to be used as a template, which researchers and PCs together edited where necessary to ensure it was fit for the purpose.

The PRIME‐UK researchers identified that the Public Involvement Impact Log in Figure 1 was a useful resource for aiding the reflective process from both the PC and researcher perspective and it was adapted by F. E. L. and E. T. to make it more suited to the PRIME‐UK research programme whereby specific rather than broad questions were used (Tables 1, 2, 3). It was adapted in this way because communication was via email rather than face‐to‐face due to the Covid‐19 pandemic restrictions. The questions asked of the PCs explored their thoughts on the changes made to documents in response to their comments. The researchers were asked about the outcomes and impact of PC involvement in the research and reflections on their own role in PPI. By adopting the Public Involvement Impact Log, it was possible to elicit reflections and PPI impacts based on the researchers' and PCs' experiences of working together on this research programme.

**Table 1:** Questions asked of three public contributors in relation to the PRIME‐UK cross‐sectional study and their responses.

Questions asked	Public contributor 1	Public contributor 2	Public contributor 3
What do you think was the outcome of your involvement, e.g., do you think there was any immediate change in what we do in response to your input?	It is impossible to judge if there is any change as a result of my input, was my immediate response. I do not have the latest versions of all the documents I commented on or those in their original form for me to compare and contrast the changes made. In addition, even allowing for Covid delaying any process it has been 12 months since you sought my input and shared some of the updated documents. It sounds a little bit harsh but I can hardly characterise this as immediate. I believe my feedback was honest, challenging and would make a difference if used. Given the number of times it is described by yourself as valuable and a previous email commenting on the value of my feedback, I'm inclined to believe that it was used and did make a difference. This is further reinforced by the fact that you have asked for my feedback again. And would like to do so and other topics. I can't believe you would be asking me if you didn't believe my feedback would make a difference hence I believe it has resulted in changes being made. Ideally, they would be more immediate and it wouldn't take 12 months to request feedback.	I appreciated that you provided some feedback for me about the changes that you made relating to my comments and that It was quite soon after I had sent them to you.	From your email of 27 April 2020, I can see that you have used some of my (and possibly others) suggestions, such as caregiver, as to how the wording on the forms could be improved. I am glad to have been able to look at the forms with ‘fresh eyes’ to help improve the design of them.
Here you could describe your thoughts on the changes we made to the documents in response to your valuable comments	It would have been great and much easier to answer the question if you had shared documents in their form before and after I (and other PwP) had provided feedback. So that any changes would have been easier to see.	*Answered this question as part of question 1*.	*Answered this question as part of question 1*.
Do you think there was any impact of your involvement, for instance, any sustained change, either positive or negative?	I find this question impossible to answer definitively based on the evidence provided.	I don't know. You have contacted me again in the next stage of your research, so that suggests that you are continuing with PPI, although it doesn't tell why. I don't think it was my involvement particularly but more a general commitment to PPI. I hope my comments are useful and encourage you to involve patients and the public in your work.	I hope there has been a positive change in the use of caregiver, which I think is a much nicer term for those who care for their loved ones. I hope that participants find when they complete the forms/questionnaires that the language is clearer to understand, that they understand what the trial hopes to achieve, that it is clearer what is being asked of them, that they are aware of the risks and what will happen to their personal information and their answers to the questions.
Did you have any reflections or thoughts on research in general and/or about the research we are doing on people with Parkinson's?	My first observation regards the speed. It appears to have taken 12 months to produce the brochure and update the consent form. Given the burden involved everyday with Parkinson's, I would suggest there would be great benefit in cutting down the timelines on the production of the literature to get people to sign up to the study. Recruitment is always a problem I know. I have actually done a literature review recruitment to Parkinson's studies and would be happy to prepare version for yourselves. I would focus on ways proven to help recruitment to studies. My second observation is perhaps a more general comment rather than a specific one given I do not know your capacity. Generally, the amount of Parkinson's research being done leaves a lot to be desired when compared to other conditions. Quite simply, more research is required. It may be not possible for you to deliver anymore by increasing capacity, i.e., headcount. I am also not sure on the other commitments that may affect the overall workload. Parkinson's research in general needs to restart as we have lost at least a year to Covid, putting back the cure to a devastating disease. This is not a Parkinson‐specific situation.	I notice that you focus on your process and things, e.g., randomisation and stages of the trial are highlighted whereas I am thinking about what the experience would be like, how it would feel. I have had to be quite proactive to find out for myself about Parkinson's and ways to get the best out of various situations so your research into providing support and education as standard for everyone diagnosed makes me feel happy. I have learned a lot from taking part in trials and from belonging to groups for people with PD.	I have many thoughts on research, especially research into Parkinson's but it would take me too long to say it all! I am just glad you are trying to improve the lives of PwP until a cure is found.
Do you have any other comments?	I am happy to help and contribute to your future projects.	I have no idea how many people volunteer for PPI opportunities, or how many you will have received comments from. I wonder if people would comment on the same or different aspects and if we agree or disagree. Or maybe it's just me, which is quite a responsibility.	Thank you.

Abbreviations: PD, Parkinson's disease; PPI, patient and public involvement; PwP, person with Parkinson's.

**Table 2:** Questions asked of three public contributors in relation to the PRIME‐UK RCT and PRIME‐UK qualitative study and their responses.

Questions asked	Public contributor 1	Public contributor 2	Public contributor 3
What do you think was the outcome of your involvement, for example, do you think there was any immediate change in what we do in response to your input?	Yes, I believe my responses were read, evaluated and actions if they added value, within a timeframe that allowed their inclusion in materials.	Thanks for your detailed feedback. It is good to know that you are making progress with your research studies. I have been able to keep in touch with a number of other people who have Parkinson's over the last months, 4 as part of a poetry workshop, 6 through exercise groups another 4 who attend an online speech and language workshop and 2 personal friends. It is clear that we are all very individual and that we experience Parkinson's in different ways. I hope that by including people with the condition in your planning that it is less likely that patients are regarded as the same, or that issues might be missed. Your detailed feedback makes me feel that my comments were carefully considered and helped to shape the detail of your study. I am pleased that you are looking at how people with Parkinson's disease can be enabled and supported to find out how we can best help ourselves deal with it and I know that many others feel the same. There are many options and it requires a lot of time and motivation to try and make sense of it all. I am looking forward to the results being shared in due course.	The only way I can tell if there was any change following my input is from the feedback from you have given me and that has been good. You have told me you value my input and where you have made changes in the vocabulary you use. You have also said that you have added questions to improve the research based on my feedback. Your feedback to my responses have been very positive.
Here you could describe your thoughts on the changes we made to the documents in response to your valuable comments	I am pleased that there has been feedback and engagement with my comments. It is first class. All to often the feedback is platitudes at best not the specific comments you have provided.	*Not answered separately*.	*Not answered separably*.
Do you think there was any impact of your involvement, for instance, any sustained change, either positive or negative?	I have no specific evidence of the results but I expect that my comments and their adoption would improve the study. I don't believe anything that would reduce the effectiveness would have been added to the already existing literature.	*Not answered separately*.	Yes, you have told me what changes and additions you have made.
Did you have any reflections or thoughts on research in general and/or about the research we are doing in people with Parkinson's?	There is still a paucity of Parkinson's research in general. The support for patients is very limited and some basic education can improve symptoms and the response to medication, As such your research into the effects of a new model of care is important as it could lead to improved standards of care.	*Not answered separately*.	Research is essential to find ways to help people to manage and improve their lives when they have a long‐term illness or disability. However, the ultimate goal in to Parkinson's research is to find a cure. I was diagnosed with Parkinson's 16 years ago and was told then that a cure was probably only 10 years away. People recently diagnosed with Parkinson's are being told the same thing: that a cure is only 10 years away. The GDNF trial I was on did not meet its primary endpoint and so was stopped but it was successful in regrowing brain cells and we, the participants, all felt the benefit. For the past 50 years, we have relied on Levadopa to get by and we should not be still saying ‘10 years’. Rant over!!
Do you have any other comments?	None.	*Not answered separately*.	I do appreciate all research that is being done to help those of us with Parkinson's to live a better life so a big thank you to you all. I am happy to help again.

Abbreviations: GDNF, glial cell‐derived neurotrophic factor; RCT, randomised control trial.

**Table 3:** Questions asked of three researchers involved in the PRIME‐UK research programme and their responses.

Questions asked	Researcher 1	Researcher 2	Researcher 3
What do you think are the outcomes of the public involvement in PRIME‐UK?	There is no doubt that the involvement of PCs in research improves the quality of the research. The involvement of PCs in the PRIME‐UK research programme has led to a number of changes: 1. The patient‐facing documents are clearer as a result. PCs guided the study team to ensure that the documents were relevant and free of ambiguity. Researchers can get bogged down in technical terminology at the expense of the clarity of the documents for the people for whom they are designed. 2. The PCs reported that they felt listened to. The researchers were very keen to feedback to the PCs to highlight where their comments were utilised, and if not, why not. It is so important to ‘close this loop’. 3. The term that we use to describe those who provide care and support for people with Parkinson's was selected by the PCs and this is now used throughout the PRIME‐UK research programme. 4. Those researchers who design patient facing documents but have no interaction with patients benefit greatly from the contribution from PCs due to a greater proximity to the patient voice.	I made several changes to the participant information booklet following the input from PCs. This included PCs providing an approximate idea of how long the questionnaires would take to complete; using clearer terminology; clarifying the options for questionnaire completion; addition of further information about data protection. We also sought their input on how they would refer to someone who supports or helps them with their Parkinson's on an unpaid basis and, while this appears to vary according to personal preference, we were able to choose a term which most individuals are likely to find acceptable. Overall, I hope that these changes have helped to ensure the study documents communicate the important points about the study as clearly as possible. Another outcome was that we established that there was a preference for face‐to‐face recruitment. While this was not feasible in this study, it added weight to our plan within the protocol to proactively contact non‐responders by telephone to answer their questions and offer support to take part; this helped us to justify the need for telephone follow‐up to the ethics committee.	In my experience, the involvement of PPI in any study is crucial as it is important to involve representatives of the sample population to ensure that we have considered/included all the issues that are important to them. It is equally important to consult them on whether they feel that the expectations that the study team have about research participants are realistic and feasible. This will then help to identify any possible barriers to study participation to ensure that the study team have the best chance of collecting the data they intend from participants while ensuring that the wellbeing of the participants is respected. By inviting feedback from PCs, the ultimate goal is to improve the quality of the research and to make it relatable to those it intends to serve.
What was the impact of the public involvement in PRIME‐UK, either positive or negative?	1. The impact for me was a sense of satisfaction that we asked those whom our research is designed to help for their input. It also reminded me of all the research that I have previously carried out where we didn't engage with PCs and how valuable this would have been. I have learned a lot in this process. 2. It is possible that the PCs feel empowered by their involvement and perhaps confident that they are doing something to help others.	It is difficult to say whether the PPI positively influenced recruitment to the study, as there is no comparison. However, it was certainly very helpful to have the honest opinions of the PCs, who themselves have Parkinson's, about the things which may make a person with Parkinson's more or less likely to take part in the study. This enabled me to rephrase some sections, add additional text and clarify the process of taking part, which I think have all improved the final participant information booklet. While one PC queried what new information this study would add, the other feedback about the study was generally positive which was encouraging.	The PCs were very frank in their feedback while being supportive of the study. On the whole, most of the suggested changes were really helpful. The PCs brought their experiences of being either someone with Parkinson's or someone who lived/cared for someone with Parkinson's. This enabled them to offer an exclusive view of what they thought was needed or where the questions could be refined in the topic guides. Interestingly, many of the suggestions/comments were shared across the differing PCs. As a result of the feedback, most of the suggestions were included. I also received some academic advice on how to revise the study design and how best to design topic guides and, while I am sure this was meant to be helpful, this was not in the brief that was sent to the PCs.
What are your reflections and thoughts on your own role in public involvement?	I have been involved in PPI for about 5 years. I believe that as a researcher, it is important that I advocate for PPI in all aspects of our research, from conception of the idea and research question right the way through to dissemination of the results. I also believe in the evaluation of this to ultimately improve the quality of the research I do. On reflection, I would have liked to have engaged a greater range of PCs in this particular activity. Not only more people with Parkinson's but their caregivers, and stakeholders such as representatives from advocacy groups.	This was my first experience of undertaking PPI activities and so I wasn't initially familiar with the process or what could be gained from it. At the time, it felt like another ‘hurdle' to negotiate before the study could be submitted to the ethics committee! However, I definitely realised the benefit of getting PPI involvement. It was really encouraging to get several prompt responses from PCs after Parkinson's UK advertised the opportunity and then to see how much time and effort the PCs had put into their responses. This PPI was conducted entirely by email and so I didn't have a particularly interactive role with the PCs. One PC asked to speak on the phone to resolve some queries about the process and this showed that there may have been some added benefit to meeting in person or on video to allow for some discussion; this may also have been helpful where the PCs had differing views on particular aspects. I am really glad that we sent the 3 PCs some feedback to explain how we had implemented their comments (even though I was a little delayed in doing this) as this made it more of a two‐way process and they appeared to appreciate knowing their contribution had made a difference.	Due to the COVID‐19 pandemic, the involvement was remote and I felt this resulted in a one‐dimensional exchange. I sent written guidance on what I wanted feedback on alongside the topic guides and invited PCs to provide comments. Although the PCs provided really comprehensive feedback, I found this a distant way of communicating, particularly as the PCs had never met me or had a chance to ask questions. It also meant that I didn't know them or have an idea of their situation and how it related to the research which is sometimes helpful to add context to the feedback. Having an email conversation removed the chance to have a discussion between the PCs and myself around the data collection. However, I think for the purposes of the task of feedback on the topic guides, this was acceptable and an efficient use of both our time. If the task was more detailed though, this format would be more difficult.
Do you have any other comments?	No thank you	The template for PCs, provided by Parkinson's UK, was useful as it listed some broad questions about both the participant information sheet and the consent form. While some of the PCs also gave other feedback, I think the template gave them a structure to follow when reviewing the documents. A particular focus of the PRIME‐UK cross‐sectional study is to recruit individuals who are typically underrepresented in research, such as those who are older, cognitively impaired, living with frailty, and so on. I am aware that the PC input we obtained may not have particularly captured the views of this group of individuals. In particular, it might have been interesting to get feedback on the information booklet for consultees to see how we could have optimised their involvement in situations in which the person with Parkinson's cannot consent to the study.	I was impressed at how engaged the PCs were in the task and the speed at which they provided feedback.

Abbreviations: PC, public contributor; PPI, patient and public involvement.

### 2.6 General reflections on the role of the public in developing this paper

**Figure 2:**
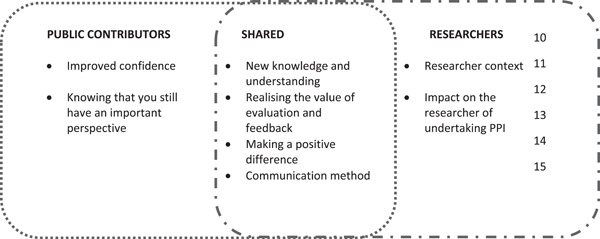
Themes from researcher and public contributor reflections (File S1) on the role of the public in the research process. PPI, patient and public involvement.

All PCs and researchers involved were subsequently invited to provide their reflections on the role of the public in research in general (Figure 2). Two of the three PCs were interested in continuing their involvement and co‐authoring a paper that reported these important reflections.^32^ The reflective process and development of themes involved regular online meetings. Eleven meetings in total were held and the PCs were paid for their attendance at these meetings at the NIHR payment rate of £25/h.^33^

The first meetings were initially focused on open discussions and reflections. Through an iterative process, we explored the key themes and highlighted important learning points for our future practices as well as learnings that could potentially benefit practices in the broader context of PPI in health research. One of the PCs involved also developed a diagram to illustrate the overlapping and distinct themes that emerged from researchers and PCs (Figure 2).

During our meetings, we reminded each other of the importance of acknowledging the challenges experienced and suggesting areas for improvement. We also discussed the paper writing process and how the PCs would like to be involved as co‐authors.

## 3 FINDINGS

### 3.1 Comments from PCs about the impact of their contribution to the PRIME‐UK cross‐sectional study, PRIME‐UK RCT and  PRIME‐UK qualitative study

Three PCs were successfully recruited to work on the studies.

The three PCs were asked to evaluate the impact of their contribution to the research (Tables 1 and 2). PCs reported that they were pleased to receive specific and detailed feedback from the researchers stating that their input was valuable. In addition, they reported that the feedback was not received in a timely manner. They stated that they perceived the repeated contact from the researchers to mean that their comments had value and were possibly impactful. They described their happiness that research is underway to improve the lives of PwP and their interest in continuing to provide feedback to researchers. Reflections from PCs on the involvement of the public in the research process, in general, are available in File S1.

### 3.2 Comments from researchers involved in the PRIME‐UK programme of research

Table 3 outlines the impact log responses from the three researchers. The researchers each expressed how valuable they had found the input from the PCs. They listed several benefits of PC input including ensuring that patient‐facing documents contained clear information in plain language, that terminology was appropriate for the target audience, and that the study procedures considered the needs of PwP. Involvement of the PCs was deemed to have led to tangible changes to the study documentation and, while it was acknowledged to be difficult to quantify the effect on study recruitment, these changes were thought to have improved the quality of the research and were anticipated to positively impact on research participant recruitment. Researcher 1 reflected on previous studies in which it would have been valuable to engage with PCs. The researchers suggested that it may have been useful to involve a wider range of PCs in this research programme and to do this earlier in the process. There was a common theme around the limitations of the use of email as the sole communication method. The importance of reporting back to PCs about the changes made because of their input was highlighted, and it was acknowledged that there was a delay in doing this. Reflections from two researchers on the involvement of the public in the research process in general are available in File S1.

### 3.3 Reflections from PCs and researchers on the impact of PPI in the research process; more commonalities than differences

Figure 2 outlines the themes that emerged from the reflections (File S1) on the impact of PPI on the research process from both PCs and the researchers. Two themes arose from PCs, two from the researchers and four were common to both groups. The PCs reported that they enjoyed the opportunities for socialisation with other PCs during the study, which led to improved confidence. They also appreciated feeling valued and knowing that their perspective is important; however, one PC also highlighted concerns that their views may not represent the views of all PwPs. Researchers reported that several factors influenced both their experience of PPI and the inclusion of PPI in the research process. These factors included their professional background, career stage and the type of research they had previously undertaken. Second, if the researcher had a previous positive experience of undertaking PPI activities, they were more likely to include PPI in their ongoing and future research. However, researchers who had limited experience in PPI reported that they lacked confidence in knowing how to involve PCs and how to use the feedback they provided.

The first theme common to both groups described the importance of providing a supportive environment for the specific patient group in question. The current project focused on understanding the challenges that PwP faces including lack of motivation, poor energy levels and slowness of thought; it was acknowledged that the timing of the day of engagement is vital. It was also suggested that caregivers could be present, not only to provide support to the PwP but to ensure that their perspective is heard. The second theme revolves around the recognition of the benefit of the evaluation of the impact of PPI on the process. Both groups acknowledged the potential negative impact of not including PPI. In addition, PCs appreciated that not all their suggestions could be implemented though communication needs to be clear to ensure that their contribution is apparent. Third, both PCs and researchers agreed that PPI engagement can make a positive difference in the research process. Fourth, and importantly, timely, personalised communication and the use of face‐to‐face communication where available, is key. To conclude, while PCs and researchers outlined their thoughts in their reflections, it is interesting to note that despite the individuals involved having different experiences, backgrounds and expertise, four of the themes were common to both groups.

## 4 DISCUSSION

At the beginning of this paper, we reviewed the current discussion relating to the evaluation of PPI; specifically, we identified a need to develop user‐friendly approaches to evaluation that provide real‐time feedback to improve practice. We have shown how the use of a simple impact log can facilitate this process. The impact log facilitated reflection and required minimal effort and resources. Furthermore, we reflected on the use of impact logs throughout the research process and are of the view that they fostered transparent and continuous communication between researchers and PCs (File S1). To the best of our knowledge, this is the first published example of how a simple tool has been used to evidence the impact of PPI in Parkinson's research. In addition, the current guidance on public involvement in Parkinson's research does not currently signpost resources that can be used to evaluate impact or suggest that evaluation should be considered from the outset. We therefore suggest that this information may be a valuable and useful added to existing Parkinson's related PPI guidance.^23^, ^34^

Our contributions to the evidence base of public involvement impact highlight the significance of not only reporting the impact on the research but also the valuable insights and lessons learned through our reflections. These reflections, we argue, are just as important in shaping our understanding and approach to public involvement, further supporting the definition of PPI Impact as detailed in the UK Standards for Public Involvement standard ‘Impact’. The impact of PPI in Parkinson's research is not widely understood and there are gaps in our knowledge of how researchers translate and apply the learnings from working with patients and the public to their practices.^12^ The involvement of PCs in the PRIME‐UK research programme provides a valuable example of the impact and benefits of involving patients and the public in health research. Through a combination of impact logs and reflections, our paper has highlighted the positive changes that resulted from working with PCs, including the ways in which they shaped the design of study materials, influenced the terminology used and improved research‐related outcomes. This evidence strengthens the existing recommendations and reports that support the positive impact of PPI in Parkinson's research.^15^, ^34^, ^35^

Furthermore, our paper has also demonstrated the many benefits of PPI. For instance, PCs expressed that they enjoyed the opportunities for social interaction and receiving support from their peers, which, in turn, enhanced their self‐confidence in offering suggestions and input to the research. That stated, the PCs were cautious and aware that their views did not represent the perspectives of all people with Parkinson's. Researchers found that working with lived experience greatly enhanced their understanding of the issues faced by people with Parkinson's. By gaining valuable insights from these individuals, they were able to ensure that their work reflected the needs and perspectives of the ultimate end users of research‐ the patient/public. Furthermore, the positive feedback that the researchers received from PCs improved their confidence in the relevance and quality of their research.

The insights and recommendations provided by the PCs have also had an impact on the researcher's perception of the positive value of public involvement in health research. These impacts were like the findings by Staley and Barron where researchers experienced ‘lightbulb moments’ and would change their values and perceptions of the issues they were studying as a result of PPI.^12^, ^36^ This collective feedback not only reinforces the existing evidence of impact but also showcases the reciprocal and diverse nature of impacts that can be achieved by involving patients and the public in health research.^5^, ^6^, ^7^, ^37^

Our work has further emphasised the importance of feedback and effective communication strategies in the context of PPI in research. To develop a better understanding of the impact and encourage continued participation, it is crucial to establish ways to provide feedback that are both meaningful and impactful to PCs. According to Mathie et al., exploring the role of feedback in PPI, a lack of feedback from researchers was found to result in decreased motivation and engagement from PCs.^38^ Conversely, providing feedback acted as a catalyst for reciprocation and promoted reflection and learning among PCs.^38^ To address these potential issues, in our future PPI practices, we aim to incorporate mechanisms for providing feedback from the earliest stage of our research processes. By doing so, we aim to avoid the negative consequences associated with a lack of feedback and foster positive relationships between researchers and PCs.

The reflections suggest that researchers would benefit from undertaking training in PPI at the earliest opportunity, especially for those who have no prior experience in PPI. To guarantee productive and meaningful public involvement, it is important that researchers are equipped with the necessary skills and tools to collaborate effectively with PCs. Our work revealed that some researchers lacked confidence due to their limited experience, causing uncertainty about how best to utilise the feedback provided by PCs. In turn, this could influence the impact that public involvement has on a study.

The identification of potential barriers to involvement reported by the PCs involved in the PRIME‐UK research programme further highlights the importance of developing plans for preferred ways of working with PCs from the outset. As a result, this approach will facilitate meaningful ways of working, overcome potential barriers to involvement and create positive experiences for all involved.

## 5 CONCLUSION

The use of tools like impact logs can facilitate reflective practice and practical improvements in the delivery of PPI. The log is easy to use for researchers and PCs. The information it collects can be used to identify valuable insights into the impact, benefits and learnings of public involvement. By reflecting on the experiences of researchers and PCs, we can recognise areas for improvement and make recommendations for future practice (see Table 4).

**Table 4:** Key recommendations based on our reflections.

Recommendation	Rationale for the recommendation
Consider using face‐to‐face and/or online approaches when working with PCs.	The researchers and PCs involved in the PRIME‐UK research programme valued the opportunity to meet and have discussions about the feedback provided. This was seen as a more meaningful and effective way of communicating when compared to receiving feedback by email.
Develop approaches to public involvement in collaboration with the PCs. Researchers should be flexible and adapt their approaches to suit the needs and preferences of the PCs to ensure inclusive and meaningful involvement.	The PCs involved in the PRIME‐UK research programme expressed challenges with involvement at times due to living with a health condition.
Researchers should aim to clearly communicate feedback to the PCs immediately after the activity that highlights the impact of their involvement on the research or for the people involved. Resources such as the UK Standards for Public Involvement, Guidance for Researchers PPI Feedback 2018 and Public Involvement Impact Logs could be used to support and plan this process.	Timely and personal feedback from researchers on the suggestions and input from PCs was beneficial for several reasons. The PCs reflected that the activity would still be fresh in their minds and this information would help them to further develop their feedback techniques and motivate them to be involved in further activities.
Consider using tools such as Public Involvement Impact Logs for continuous reflection and for identifying areas to improve future PPI practices.	The use of Public Involvement Impact Logs demonstrated how they could be used in practice to identify where future practices could be further improved.
Provide evidence of impact: Consider using existing tools such as the Public Involvement Impact Log.	The PCs suggested that providing a ‘before and after’ version or documents would be useful evidence of the impact of their involvement. The Public Involvement Impact Log would capture the changes made to documents following the suggestions from PCs.
Consider working with organisations and charities in your area of research who may be able to provide support with the recruitment of PCs and advertisement of opportunities.	Parkinson's UK supported the recruitment of PCs for the PRIME‐UK research programme, which resulted in several responses from interested individuals.
Dedicate spaces for peer support and socialising: Researchers should consider building in time for socialisation and peer support opportunities to maximise the benefits of public involvement for the PCs.	The PCs involved in the PRIME‐UK study reported that they enjoyed the opportunities for socialising and peer support, which was an unexpected benefit of their involvement.

Abbreviations: PC, public contributor; PPI, patient and public involvement.

## AUTHOR CONTRIBUTIONS

Fiona E. Lithander, Emma Tenison and Noreen Hopewell‐Kelly devised the project. Fiona E. Lithander, Emma Tenison and Carmel McGrath communicated with the public contributors. All authors together conducted the data analysis and wrote the manuscript. All authors commented on and agreed on the final manuscript.

## CONFLICT OF INTEREST STATEMENT

The authors declare no conflict of interest.

## ETHICS STATEMENT

As per the Health Research Authority/NIHR INVOLVE statement, ethical approval was not required for this patient and public involvement piece.^29^

## Supporting information

Supporting information.

## Data Availability

All data generated or analysed during this work are included in this published article.
