# Supplementary material for: Working with public contributors in Parkinson's research: What were the changes, benefits and learnings? A critical reflection from the researcher and public contributor perspective
Source: Health Expect. 2023 Nov 21;27(1):e13914. doi: 10.1111/hex.13914 (PMC10768872; doi:10.1111/hex.13914)
Supplement: Supplementary file 1 — Supporting information. [file HEX-27-e13914-s001.docx]

**Supplementary File 1**

### Reflection from PC 1 (M, age 56y, with 6 years of experience in PPI).

I was interested in working on this study as I believe patients have unique insight which can improve research so, when opportunity arises, I feel compelled to participate, so that researchers and patients can move forward in the best way possible. Throughout my involvement in this research, I have been involved in reviewing the terminology and providing feedback on the study documentation including the participant consent forms and study brochures. I had different experiences relating to the feedback process and here I reflect on these experiences in two main areas; firstly, the ways in which I provide feedback as a public contributor and secondly how and when feedback is provided by study teams.

When providing feedback, I always try to ensure that my feedback is honest and challenging to ensure that as much as possible, my suggestions make a positive difference. However, it is difficult to know if you’ve provided feedback in the ‘right’ way or if you’ve made a valuable contribution without someone communicating this information back to you. From my own personal experience, I found it encouraging to receive positive feedback on several occasions via email that my contributions were valuable and used. For example, I received the following email from a research team member when I reviewed their terminology: “Firstly, thanks again for your help earlier this year in reviewing documents and helping us decide on use of terminology such as carer versus care giver.  Your input was very helpful, probably more than you realise”. As a result of this email, I was confident that my suggestions were useful, and this was further reinforced when the team asked me on several occasions afterwards for my ongoing feedback and support.

#### Top tips for researchers and public contributors from PC1

It is important as a public contributor to present your suggestions in a fair but challenging way- ask the researchers questions and provide your honest opinions. It might seem challenging at first to be critical but the aim of providing your help and support is to ensure the research is patient friendly, accessible, and relevant. My tips for researchers would be to provide feedback to public contributors about the difference they have made- even if it is a small difference. It is important to be open and honest to public contributors- if you haven’t used their feedback that’s fine but it’s important to let the public contributors know why it hasn’t been used. Any feedback from researchers provides helpful reassurance when, as a public contributor, you might be questioning yourself and whether you have helped the study or not. It is important for researchers to consider *how* they show public contributors the difference they’ve made. For example, one approach might include providing a ‘before’ and ‘after’ highlighting how the ‘after’ version has changed following the input from public contributors.

It is important for researchers to think about *when* they will provide feedback to public contributors. Sometimes because research teams are busy it can take a while for them to get in touch to let public contributors know the outcome of their involvement. From the perspective of a public contributor, it is important to us that we receive feedback (in some format for example via email or a phone call) as soon as possible. This timely feedback is useful as we have just undertaken the activity therefore it is fresh in our minds, but it also helps us to further develop our feedback techniques and motivates us to become involved in further activities. I’ve enjoyed my time working on the research project and I am glad that I have made a difference which I hope will mean that the study participants have a positive experience, and the study has produced meaningful outcomes.

#### Reflections on using the Public Involvement Impact Log, from PC 1

The impact logs were simple to use requiring no specialist knowledge to complete, making them highly accessible. The physical completion was not time pressured taking the pressure off for people struggling with dexterity. The logs provided a framework and focus to the engagement. The distribution and the sharing of the logs acted as a signal that the communication with the public contributors were being taken seriously.

### Reflection from PC 2 (F, age 69y, with 7 years of experience in PPI)

My journey into public involvement started when I was a participant in two Parkinson’s research projects. As a result of participating in these studies I developed a better understanding of Parkinson’s as well as a more positive attitude to living with Parkinson’s. I wanted to help make it easier and more enjoyable for others to participate in research, so I started volunteering for Parkinson’s UK.

I expressed interest in PRIME-UK research programme and continued to be involved because it was a project that I felt would benefit everyone with Parkinson’s by providing specialist clinical services and information that are going to be relevant to people’s needs. This is particularly important for people living with Parkinson’s as it affects people in so many ways. I liked the idea of working on a project that I know will benefit people in the immediate future and was designed to meet the individual needs and preferences of people living with Parkinson’s.

#### What have been the benefits of being involved? From PC2

Throughout my time working on the PRIME-UK research programme there have been aspects of my involvement that I have particularly enjoyed. For example, I have been asked to read and comment on documents to ensure that they are in plain English and can be understandable to wider audiences. I also liked the prospect of being able to directly shape the PRIME- UK intervention at the later stages of the study. In one activity, I was asked to consider the treatment plan that would eventually be delivered to patients and suggest changes to this plan. For this activity, my lived experience was crucial as I had to consider how others with Parkinson’s would react to the treatment plan. In some areas of research that are patient-facing researchers may not fully recognise the added benefit of undertaking public involvement in addition to their daily interactions with their patients, their families, and carers. Researchers who work in non-patient facing roles such as laboratories may be able to imagine more clearly the advantages of working with patients and the public to develop and design research to ensure its benefit and relevance to the service users. I believe that my involvement in the PRIME-UK research programme has shown researchers (even those who are clinicians and may interact with patients daily) the importance of working with patients and the public as partners in research regardless of the nature of the research and roles of the researchers.

#### What have been the challenges and what has been learnt? From PC2

Being involved as a person living with Parkinson’s can, at times, make contributing difficult. With this health condition, everything is slower including your thinking, speaking, and typing. At times, it can be challenging to remain motivated and there are times of the day when it is difficult to find the drive or energy to do anything. If I am having to self-motivate myself to get involved and seek opportunities to be involved, this is even harder during these more challenging times. Having long periods between public involvement tasks adds to this; therefore, I would suggest that if researchers are working with people who are living with a health condition, they build in time to have initial conversations at the earliest stage to establish how and when the public contributors would like to be involved.

I also find myself apprehensive when expressing my opinions as they are my personal opinions and not representative of all people living with Parkinson’s. I would recommend that researchers ensure public contributors are made aware that their views and suggestions are valuable contributions to an overall discussion and development of a research project, with the aim to create research outcomes that are going to be considered relevant to those affected by the research.

It is my hope that engaging in discussions in a structured way helps the researchers think through the questions that need asking, to ask and listen to responses of the public contributors and be less likely to put difficult issues aside without proper consideration.

As a result of my involvement in the PRIME-UK research programme, I have also had opportunities to learn and discover new things. For example, how PhD projects are used to further research into Parkinson’s. To my knowledge, there are a variety of health professions that are involved in treating and supporting Parkinson’s patients for example speech and language therapists, physiotherapists, neurologists and specialist nurses. I believe that these healthcare providers all have an important role in caring for people with Parkinson’s and I’d be interested to learn if research into Parkinson’s has been undertaken by these allied healthcare professionals and something that I’d like to see in the future would be a collection of research and outcomes they’d produced.

I have valued my time working on the PRIME-UK research programme and I have enjoyed the peer support opportunities. I like to talk things through with others as this helps me gather my thoughts, so being involved with groups and having friends with Parkinson’s has helped me feel more confident in contributing. The research team have created accessible and flexible opportunities for me to be involved, for example I have been able to share my thoughts over Zoom. The team have been responsive to my suggestions by being open to discussion and making visible changes based on my recommendations. It has been a rewarding experience and receiving acknowledgement and feedback has encouraged me throughout my journey on the PRIME-UK research programme.

#### Reflections on using the Public Involvement Impact Log, by PC2

Answering the questions from the Impact log about our involvement with the PRIME-UK researchers was easier for me to do than starting with a blank sheet of paper. The log asked mostly open questions and acted as a prompt, and I felt comfortable giving my opinions.

### Reflection from Researcher 1 (F, age 47y, 5-10 years prior experience of PPI)

Given that I have worked as a research scientist for over 20 years, I find it hard to understand how it is only in the last 6-7 years that the importance of involving the public in my research is on my radar. One reason may be that early in my career, my research involved increasing our understanding of biological mechanisms such as appetite regulation in healthy individuals, and the involvement of the public was not warranted. The type of research that is undertaken and indeed whether it includes the study of a particular patient group determines whether a researcher deems it necessary to include the public. As my career progressed and included undertaking more research in people with different diseases, it became clear to me that discussion with PCs should be an integral part of all clinical research programmes from conception through to the dissemination of the results. Public contributors, including those with the disease under study have so much experience which is of immense value to any research programme and can help inform key research questions. Indeed, other stakeholders, including advocacy groups, caregivers and family members should and could be invited to contribute

I was first introduced to PPI in 2016 and it was such a revelation. We engaged with people who had a specific disease to ascertain their views on research questions and methods, listened to their views, and subsequently integrated these into our research endeavours. How novel this was to me! I remember one instance where we engaged with the public about a project we had in mind. Their input suggested that the proposed methodology was nonsensical and based on that feedback, we decided not to pursue that project any further. This was incredibly helpful for me to strengthen my understanding of the importance of involving the public. In the PRIME-UK research programme, not only was I keen to engage with members of the public, but with people with Parkinson’s, to bring their wealth of knowledge into the research programme.

In PRIME-UK, the impact log was useful as it allowed us to provide structure not only in our communication with PCs but also with other researchers. I regret that there were significant delays in our communication with PCs. I also regret that all communication was via email and that we did not have the opportunity to meet in person. In hindsight, we could have sent the revised PILs to the PCs to allow them to see where changes were made from their suggestions. In future, I will prioritise face-to-face meetings with PCs. I also endeavour not only to include PPI in each future research programme that I work on, but to evaluate the impact of this in a timely manner and encourage others to do the same.

### Reflection from Researcher 2 (F, age 34y, no prior experience of PPI)

At the start of my PhD, when I was designing the PRIME-UK cross-sectional study, I was advised by my supervisors to seek input from a PPI group. As a clinician, I was familiar with the concept of shared decision-making between healthcare professionals and patients, as well as patients helping to shape clinical services and improve patient experience. It therefore made sense to me that it would be important to involve people living with Parkinson’s in the design of the research and study documentation, although I was not familiar with the term ‘PPI’ and did not know how to approach doing this or how to evaluate its impact. Fortuitously, a seminar was subsequently advertised to postgraduate research students providing an overview of the topic. I was also grateful for the support of Parkinson’s UK in advertising the public involvement opportunity and for providing us with guidance on questions to ask the PCs.

The PCs improved the readability of the documents, however, some of their feedback suggested that they had misunderstood the study requirements or procedures slightly, demonstrating that I needed to convey this information more clearly. It was so helpful to have the PCs’ perspective because, when you are ‘caught up’ in the study design and delivery, it can be hard to appreciate how the information will seem to someone reading about the study for the first time. I found it difficult to know how to deal with comments that could not be implemented, for example the preference for face-to-face recruitment, since the PRIME-UK cross-sectional study was designed to be delivered without in-person visits. I learnt that it isn’t always possible to incorporate every comment or suggestion.

All PPI activity was conducted by email. This had the advantage that the PCs could review documents in their own time, without the need to travel. However, where a particular point was raised in the feedback from only one PC, it would have been interesting to get the views of the other PCs on this aspect. Where differing views were expressed, it would have been useful to discuss this to reach a consensus. One PC phoned to check that they were ‘doing it correctly’, as they were concerned that their responses may not make sense to the researchers. These concerns could have been more easily allayed if the PPI was carried out as a face-to-face workshop. Although we responded to each PC individually to outline the changes made in response to their feedback, a few weeks elapsed before we managed to do so; if we had gathered the feedback in-person or on a video call, it would have been easier to feedback immediately about how we might incorporate their comments and, if it was not feasible to implement a suggestion, explain why this was the case.

One of our questions was around the preferred terminology to use for someone who informally supports someone with Parkinson’s. All the PCs lived with someone who provided some degree of support with whom they could discuss this point, which proved to be extremely helpful. Whilst we settled on the term ‘caregiver’ to refer to these participants, the feedback prompted me to explain throughout the documentation that not everyone who lives with or supports someone with Parkinson’s would consider themselves to be a ‘caregiver’. I will be mindful of the need to be sensitive around this terminology in my future clinical and research activities.

We could have involved PCs earlier in the process of designing the study. This could have included asking for their input on which symptoms and domains to measure in the PRIME-UK cross-sectional study, as well as involving them in piloting the questionnaires to assess burden/length. This study also recruited adults lacking capacity to consent to research. In retrospect, it would have been helpful to directly involve individuals who supported someone with, for example, dementia, to get their feedback on the information booklet for potential consultees, which may have provided insights into the information needed by proxy decision-makers.

The PCs were pleased to hear from us about the changes made in response to their feedback and said that researchers do not always do this, showing me that this is a crucial part of the process. Asking the PCs to complete the impact log was a straight-forward and helpful way to gauge what they thought was the outcome of their involvement, although I recognise that it would have been preferable to share the impact log sooner after the activity. The impact log responses highlighted that, despite our efforts to give feedback about the changes made, there was still uncertainty as to whether their comments and input had made a difference. There is scope to improve this process so that PCs are informed of the outcomes of their involvement and understand how it led to sustained change.
